# The effectiveness of obturation materials on pulpectomy outcomes in primary teeth: a retrospective study

**DOI:** 10.2340/biid.v13.47015

**Published:** 2026-10-20

**Authors:** Penmatsa Chaitanya, Gontla Devi Saranya, Peddi Ravigna, Chandrappa Vinay, Kadidal Shamanna Uloopi, Gadisetti Lakshmi Surekha

**Affiliations:** Department of Pedodontics, Vishnu Dental College, Bhimavaram, India

**Keywords:** calcium hydroxide, iodoform, pulpectomy, root canal filling materials, zinc oxide-eugenol cement

## Abstract

**Objective:**

Pulpectomy is an essential procedure for preserving primary teeth with irreversible pulpitis. Its success may vary according to lesion etiology, preoperative pulp condition, follow-up duration, obturation materials, restorations, and other clinical factors. This study aimed to evaluate the success of pulpectomy in primary teeth treated with different obturation materials.

**Materials and methodology:**

Records of children aged 4–10 years who underwent pulpectomy were retrospectively retrieved and assessed using clinical and radiographic records. The success rate was determined by considering clinical and radiographic findings together. Resorption of obturation materials was assessed radiographically at 3, 6, 9, and 12 months by comparing the follow-up radiographs with the immediate post-treatment radiographs. Data were analyzed using the chi-square test.

**Results:**

The study evaluated 225 pulpectomised primary teeth using Endoflas (*n* = 85), Metapex (*n* = 73), and zinc oxide eugenol (ZOE) (*n* = 67) as obturating materials. The success rates were comparable among the groups at 3 months. Significant differences were observed at 6 and 9 months (*p* < 0.05), with ZOE showing the highest success rate, followed by Endoflas and Metapex. At 12 months, a significant difference in success rate was also observed among the groups (*p* = 0.001) with ZOE still demonstrating the highest success rate (92.5%), followed by Endoflas (80%) and Metapex (42.5%). Rate of resorption differed significantly among the materials (*p* < 0.001), with Metapex showing the highest resorption rate at 6 months (29.9%), 9 months (43.8%), and 12 months (75.3%), followed by Endoflas and ZOE.

**Conclusion:**

Pulpectomy success rate was found to be highest when ZOE was used as the obturation material, followed by Endoflas and Metapex, at the 6, 9 and 12 months of follow-ups. Metapex exhibited the highest resorption rate among the three materials.

## Introduction

Maintaining primary teeth in a functional state until their natural exfoliation is essential for preserving the integrity and function of the developing dentition [1]. Untreated irreversible pulpitis or pulpal necrosis in primary teeth may result in pain, difficulty in mastication, and pathological changes that can affect the underlying permanent successor [2]. The American Academy of Pediatric Dentistry (AAPD) recommends pulpectomy for the treatment of primary teeth with irreversible pulpitis or necrotic pulp when preservation of the tooth is indicated [3]. However, pulpectomy in primary teeth can be challenging because of the complex and tortuous root canal anatomy and the close relationship between the roots of primary teeth and their permanent successors. Consequently, the properties and behavior of the obturation material are important considerations in the outcome of treatment [4].

Zinc oxide eugenol (ZOE) has been widely used for root canal filling in primary teeth since its introduction by Bonastre in 1837. Eugenol possesses analgesic and anti-inflammatory properties [5]. However, ZOE may persist in periapical tissues when extruded beyond the root apex because its resorption may be slower than physiological root resorption of the primary tooth [6]. To address this limitation, alternative obturation materials, including calcium hydroxide–iodoform pastes such as Metapex (Meta Biomed Co., Ltd., Cheongju, South Korea) and Vitapex (Neo Dental Chemical Products Co., Ltd., Tokyo, Japan), have been introduced. These materials are readily resorbed when extruded beyond the apex and are relatively easy to use [7].

Another alternative is Endoflas F.S. (Sanlor Laboratories, Colombia), which contains ZOE, iodoform, and calcium hydroxide, along with radiopaque components. The combination of these components provides antibacterial and resorbable properties together with the sealing characteristics of ZOE. Endoflas has demonstrated antibacterial activity against several microorganisms, including Enterococcus faecalis [8]. Its resorption characteristics differ from those of other obturation pastes, as material extruded beyond the root apex is reported to resorb, whereas material retained within the root canal is relatively resistant to resorption. This characteristic may help reduce the development of an intracanal hollow-tube effect [9]. Endoflas is also considered biocompatible and resorbable, with extruded material eliminated through phagocytosis.

Previous studies have reported variable success rates for these obturation materials across different follow-up periods. ZOE has demonstrated success rates ranging from 85 to 100% [10–12], while reported success with Endoflas has ranged from 70 to 100% [13, 14]. Metapex has also shown variable outcomes, with reported success as low as 58% [2]. Differences among studies may be related to variations in study design, sample characteristics, follow-up duration, clinical conditions, and outcome assessment criteria. Moreover, relatively few retrospective studies have compared Endoflas, Metapex, and ZOE within the same clinical population. Therefore, this study aimed to retrospectively evaluate the success of pulpectomy using these three obturation materials in primary teeth of children aged 4–10 years and to assess their respective obturation-material resorption patterns.

## Materials and methods

This study was carried out at Vishnu Dental College, Andhra Pradesh, India. The pulpectomy procedures were performed by Pediatric Dentistry residents under faculty supervision, according to the AAPD guidelines and under rubber dam isolation. The Institutional Scientific Review Board and Ethical Committee (IECVDC/24/PG01/PPD/IVT/71) approved this retrospective study in accordance with the ethical principles of the 1964 Declaration of Helsinki and its subsequent revisions. Data were retrieved retrospectively from the institutional electronic medical record system from June 2026 to July 2026, and records from January 2021 to March 2026 were screened. Primary anterior and molar teeth in children aged 4–10 years that had undergone pulpectomy, with available pre- and post-treatment radiographs, and followed clinically and radiographically at 3-month intervals for a minimum of 1 year were included. Children with systemic diseases affecting oral hygiene maintenance or incomplete clinical records were excluded.

A summary of the data collection process is presented in Figure 1. All data were retrospectively extracted from institutional records and corresponding clinical and radiographic findings were recorded using a structured data extraction form. The collected information included demographic characteristics (age and sex), initial visit date, presence or absence of periapical of inter-radicular radiolucency, type of root filling material (Endoflas, Metapex, or ZOE), and type of coronal restoration (glass ionomer cement [GIC], composite, or stainless-steel crown [SSC]). Follow-up records were reviewed for visit dates, chief complaints, and findings from clinical and radiographic examinations. Clinical findings included oral hygiene status, discomfort, restoration integrity, tenderness to percussion, tooth mobility, and the presence of sinus tract/fistula or other soft-tissue pathology. Radiographic findings included periapical and interradicular radiolucency, pathological internal or external root resorption, and resorption of the root canal filling material. For teeth that were lost during the follow-up period, the date of loss and associated clinical symptoms were documented.

**Figure 1:**
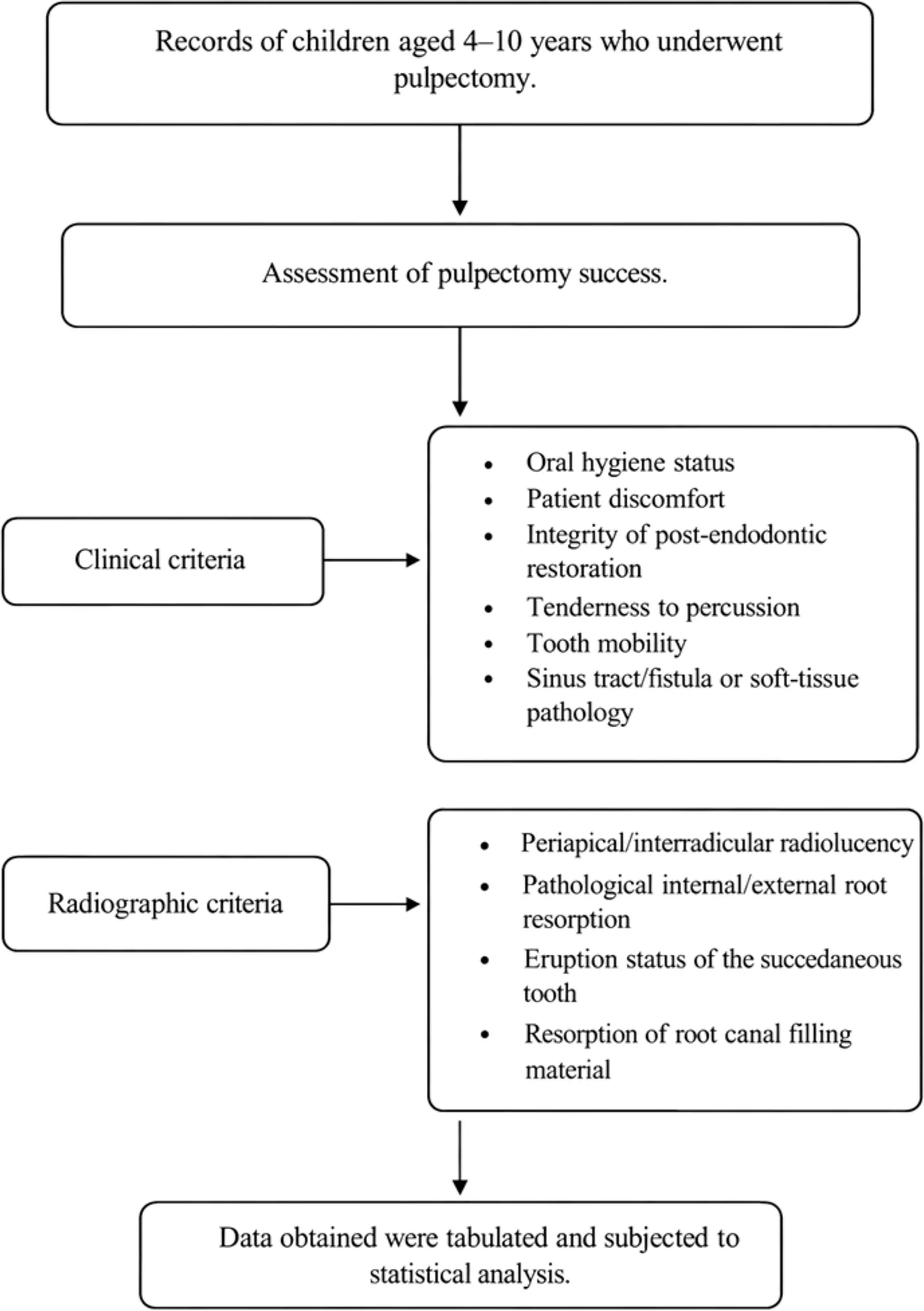
Methodology of data collection.

Pulpectomy treatment was considered successful when there were no signs of gingival disease, discomfort, abnormal mobility, failure of the coronal restoration requiring extraction or retreatment, new periapical or interradicular radiolucency, persistence or progression of a pre-existing lesion, or pathological internal or external root resorption. The treatment was considered a failure if any of these findings were present. Both clinical and radiographic evaluations were performed by a single evaluator who was blinded to the type of root filling material used. A summary of the study characteristics is illustrated in Table 1.

**Table 1:** Distribution of study variables and findings.

S. No	Variable	Finding	*N* (%)
1	What is the obturation material used?	Endoflas	85 (37.8)
		Metapex	73 (32.5)
		ZOE	67 (29.7)
2	Oral hygiene maintenance	Yes	190 (84.4)
		No	35 (15.6)
3	Are there any signs of discomfort for the patient?	Yes	15 (6.7)
		No	210 (93.3)
4	Is the post endodontic restoration (GIC/composite/stainless steel crown) intact?	Yes	174 (77.4)
		No	5 (2.3)
5	Is the tooth tender on percussion?	Yes	5 (2.3)
		No	220 (97.7)
6	Is the tooth mobile?	Yes	17 (7.6)
		No	208 (92.4)
7	Is there sinus tract/fistula or any pathology on soft tissues surrounding the tooth?	Yes	5 (2.3)
		No	220 (97.7)
8	Is peri/inter radicular radiolucency present?	Yes	34 (15.2)
		No	191 (84.8)
9	Are there any signs of internal/external root resorption?	Yes	57 (25.4)
		No	168 (74.6)
10	Is there resorption of root canal filling material?	Yes	64 (28.5)
		No	161 (71.5)
11	Is the succedaneous tooth close to eruption?	Yes	72 (32)
		No	153 (68)

GIC: glass ionomer cement.

Natural exfoliation of primary teeth was categorized as treatment success, whereas premature tooth loss or delayed pathological root resorption following treatment was considered failure. The occurrence of early tooth loss or delayed root resorption was assessed by comparing the treated primary tooth with the contralateral tooth and/or adjacent primary teeth that had not undergone pulpectomy. In cases where contralateral or adjacent untreated teeth were unavailable, the developmental stage of the permanent successor was considered for assessment.

The resorption of the obturation materials was assessed radiographically at each follow-up interval (3, 6, 9, and 12 months). Resorption rate was defined as the percentage of treated teeth showing radiographic evidence of reduction or disappearance of the obturating material compared with its appearance immediately after treatment.

## Results

Obtained data were tabulated using Microsoft excel, all qualitative variables were recorded in frequencies and percentages and compared using chi-square test using SPSS software version 27.0. A probability value of *p* ≤ 0.05 and *p* ≤ 0.000 was considered for statistical significance and high significance, respectively.

A total of 76 children underwent pulpectomy in 225 primary molars, with a male predominance (64%). Most patients (84.4%) maintained good oral hygiene. Clinically, 93.3% showed no discomfort, 97.7% had no tenderness on percussion, and post-endodontic restorations were intact in 77.4% of cases. Radiographically, peri/inter-radicular radiolucency was observed in 15.2% of cases, and root resorption in 25.4%. Among the obturation materials, Endoflas was used in 37.8% of teeth, Metapex in 32.5%, and ZOE in 29.7% (Table 1).

At the 3-month follow-up, no statistically significant difference in success rates was observed among the three obturation-material groups (*p* = 0.351). A statistically significant difference was observed at 6 months (*p* = 0.021), with success rates of 94.1% for Endoflas, 89% for Metapex, and 100% for ZOE. At 9 months, the difference in success rates among the groups was highly significant (*p* < 0.001), with rates of 91.8, 65.8, and 98.5%, respectively. At 12 months, a statistically significant difference was also observed among the groups (*p* = 0.001), with success rates of 80% for Endoflas, 42.5% for Metapex, and 92.5% for ZOE (Table 2).

**Table 2:** Comparison of success rates of pulpectomy of the three obturation materials.

Follow-up period	Material	Success rate (%)	*P*
3 Months	Endoflas	100	0.351
	Metapex	98.6	
	ZOE	100	
6 Months	Endoflas	94.1	0.021*
	Metapex	89	
	ZOE	100	
9 Months	Endoflas	91.8	0.000*
	Metapex	65.8	
	ZOE	98.5	
12 Months	Endoflas	80	0.001*
	Metapex	42.5	
	ZOE	92.5	

ZOE: zinc oxide eugenol.

* Significant; *p* < 0.05, chi-square test.

Resorption patterns also differed significantly among the materials. At the 3 months follow-up, no resorption was observed with any of the materials. After 6 months, ZOE showed no resorption, while Metapex showed the highest resorption rate of 29.9% (*p* = 0.000). By 9 months, resorption rate was still highest with Metapex (43.8%), followed by Endoflas (12.9%) and ZOE (0%) (*p* = 0.000). Similarly, at 12 months, Metapex showed the highest resorption rate (75.3%), followed by Endoflas (20%) and ZOE (3%) (*p* = 0.000) (Table 3).

**Table 3:** Comparison of resorption rates of obturation materials at different time intervals.

Follow-up	Material	Resorption rate (%)	*P*
3 Months	Endoflas	0	-
	Metapex	0	
	ZoE	0	
6 Months	Endoflas	2.4	0.000*
	Metapex	21.9	
	ZoE	0	
9 Months	Endoflas	12.9	0.000*
	Metapex	43.8	
	ZoE	0	
12 Months	Endoflas	20	0.000*
	Metapex	75.3	
	ZoE	3	

ZOE: zinc oxide eugenol.

* Significant *p* < 0.001, Chi-square test.

## Discussion

The perfect pulpectomy obturation material should be resorbable, have antimicrobial qualities, and not affect developing tooth buds or periapical tissues. It must also be radiopaque, adhere to the walls, not shrink, be simple to remove if necessary, be easy to fill into the canals, and not cause tooth discoloration. However, none of all the obturating materials available meet all of these requirements [5]. Until 2008, ZOE was the only material specifically suggested in the clinical guidelines created by the AAPD, and it has long been the preferred material for filling the root canals of deciduous teeth. The AAPD guidelines started including iodoform-based pastes as viable substitutes for ZOE in 2009, based on published studies [1]. Endoflas satisfies most of the necessary characteristics of an optimal root canal filling material for primary teeth since it is a combination of zinc oxide, eugenol, calcium hydroxide, and iodoform.

Over a 12-month period, this study assessed the success rates of three obturation materials in pulpectomized primary teeth: ZOE, Endoflas, and Metapex. Previous studies have assessed clinical and radiographic findings as complementary measures of pulpectomy success rates [15–19]. Although clinical signs and symptoms may remain absent, radiographic findings, including persistent or newly developed periapical or interradicular lesions or pathological root resorption, may indicate treatment failure. Therefore, in this study, clinical and radiographic findings were considered together to determine the treatment success. The progressive reduction in overall success during follow-up, particularly at 9 and 12 months, emphasizes the importance of continued clinical and radiographic monitoring following pulpectomy. Although radiographic healing may occur over several months, longer follow-up is important for detecting delayed healing, pathological changes, and late treatment failure.

Eugenol’s anti-inflammatory and analgesic properties have been attributed to the 60% that is released in the periapical zone just after obturation, which drops to 40% by the next day and to nil after a month [5]. In this study, ZOE had a 92.5% success rate at the 12-month follow-up period. As an obturation material, ZOE has been the subject of conflicting success rates in the literature. According to Barr et al. [20], 62 primary molar pulpectomies that were followed up on for 40.2 months had an overall success rate of 82.3% using ZOE. During a 9-month follow-up, Nadkarni and Damle [21] found that 70 primary molars from 60 children ages 4–8 had an 88.5% success rate using ZOE. Similarly, a systematic review reported ZOE success rates ranging from 85 to 100% [9], while a retrospective study by Sadrian and Coll [11] reported approximately 82–84% success at longer follow-up periods. These variations may reflect differences in study design, sample size, treated teeth, initial pulp condition, follow-up duration, and outcome assessment criteria. Prior investigations have documented ZOE failure rates of 11–35%, similar to our observed decline over time [22]. Ou-Yang et al. reported comparable overall success rates for ZOE and Metapex at 12 months, with success rates of 85.9 and 82.9%, respectively [12].

ZOE sets into a dense mass that provides an effective seal against bacterial leakage, while the eugenol component offers antibacterial and anti-inflammatory effects that aid periapical healing. These factors contribute to the success of obturation with ZOE material. Unlike materials such as Metapex, that resorb too quickly within the canal, ZOE resorbs more slowly, maintaining stability and preventing voids that could harbor reinfection, though extruded particles may persist beyond the apex [9]. These might be the causes of the increased ZOE cement clinical and radiographic success rates seen in this study.

Endoflas showed an overall success rate of 80% at 12 months in our study. Similarly, Rewal et al. [23] reported 100% overall success in primary molars over 9 months, whereas Fuks et al. [13] reported 70% success over 6–52 months, reflecting variability among studies. Despite this variation, Endoflas has been recognized for its healing ability, bone regeneration characteristics, and resorption of excess material without producing a hollow-tube effect within the root canals [12].

Metapex in this study showed a significant decrease in success over the study period, with a success rate of 42.5% at 12 months of follow-up. The antibacterial qualities of Metapex and its unique ability to rapidly resorb from periapical tissue are linked to the success of pulpectomy with Metapex in primary teeth. Metapex, a calcium hydroxide-based paste was hence used to obturate teeth in this study, that are nearing exfoliation or have furcation radiolucency in primary teeth. However, as the resorption of Metapex is faster than the primary root, a hollow tube effect due to resorption of material in the root canal can lead to occurrence of re-infection [24]. This might be one of the reasons for decreased success rates of Metapex obturated teeth. Similarly, an *in vivo* study by Gupta and Das reported an overall success rate of 58% for Metapex over a 9-month period, indicating comparatively lower short-term success [2]. In contrast, Subramaniam and Gilhotra reported no significant difference in overall success among ZOE, Endoflas, and Metapex after 18 months, suggesting that the observed differences in success rates may vary according to study characteristics and follow-up duration [25].

In this study, post-endodontic restorations (GIC/composite/SSC) were lost in 51 teeth, of which 40 (78.5%) were associated with treatment failure. Complete cavity closure following treatment is essential for the effectiveness and durability of endodontic treatment [26]. After endodontic treatment of primary molars, a post-endodontic restoration provides a coronal seal and helps prevent microbial penetration over time. Loss or fracture of the restoration may allow microbial infiltration and contribute to endodontic treatment failure [27].

The resorption rates observed in this study further support the clinical findings. After 3 months, ZOE consistently exhibited the lowest resorption, whereas Metapex showed rapid resorption, and Endoflas demonstrated intermediate resorption rate. At 12 months, ZOE exhibited the lowest resorption rate of 3%. These results are consistent with those reported in a systematic review conducted by Barcelos R et al. [10], who concluded that ZOE resorbs at a rate compatible with the physiological resorption of primary teeth, thereby maintaining the integrity of the obturation over time without forming voids.

Endoflas showed a resorption rate of 20% at 12 months, which is higher than that of ZOE but were within acceptable limits. The extruded Endoflas material was found to clear faster than other materials, preserving canal integrity [9] Brar et al.’s 2019 *in vivo* study [24] examined the relationship between root resorption and the obturation quality of ZOE, Endoflas, and Metapex. While Metapex had the hollow tube effect in most of the cases (100%) at the 12-month follow-up, Endoflas had the highest success rate with the least incidence of resorption both inside and outside the root canal. However, in this study, Metapex reached a resorption rate of 75.3% at 12 months, exceeding physiological root resorption. The rapid resorption of Metapex leads to voids within the root canal system, increasing the risk of reinfection and treatment failure. Similar patterns of rapid Metapex loss correlating with pulpectomy failure have been reported by Chen et al. [6].

ZOE is still an effective obturation material for primary teeth, according to the study’s findings, providing long-term clinical and radiographic success with minimal resorption. Endoflas presents a viable alternative, particularly in cases where overfilling is a concern, due to its favorable resorption characteristics and antimicrobial properties. Metapex, while initially effective, may not provide long-term success due to its rapid resorption, which can lead to treatment failure. Limitations of this study include its retrospective study design, the fact that treatment protocols and operator variability could not be fully standardized, and a follow-up period limited to 12 months. Future considerations are randomized controlled trials (RCTs) with larger sample sizes can provide stronger evidence.

## Conclusion

The success rate of pulpectomy was higher when ZOE was used as obturation material compared to Endoflas and Metapex. Metapex demonstrated rapid resorption compared to Endoflas and ZOE. These findings suggest that understanding the resorption patterns of these materials is essential to prevent treatment failure, especially in growing children with physiologic root resorption. Since pulpectomy is one of the most frequently performed procedures in pediatric dentistry, insights from this study can improve treatment planning and material selection for the treatment procedures.

## Data Availability

The data related to this study are available from the corresponding author upon reasonable request, subject to appropriate ethical and institutional restrictions.
